# A multi-factorial analysis of hypotension after intubation for patients with hypertensive intracerebral hemorrhage

**DOI:** 10.3389/fmed.2026.1843544

**Published:** 2026-06-26

**Authors:** Wankang Dian, Wenkai Zhang, Shouzhi Fu, Luyu Yang

**Affiliations:** Department of Emergency/Intensive Care, Third Hospital of Wuhan, Wuhan, China

**Keywords:** hypertensive, hypotension, intracerebral hemorrhage, multi-factorial, tracheal intubation

## Abstract

**Objective:**

To investigate what factors predispose patients with hypertensive intracerebral hemorrhage to develop hypotension after intubation, and to use this knowledge to direct treatment efforts away from hypotension and prevent further ischemia caused by it.

**Methods:**

This was a single-center, retrospective observational study. Of 451 patients with hypertensive intracerebral hemorrhage admitted to our emergency department between January 2019 and September 2022, 275 met the eligibility criteria and were included. Those patients were divided into hypotensive group (124 cases) and non-hypotensive group (151 cases) according to their blood pressure changes after intubation. The analysis compares the demographic data, vital signs, and laboratory results of the two groups of patients. Based on the comparison analysis, Logistic regression method was used to analyze indicators with statistical differences to determine the correlation between the selected variables and drop of blood pressure.

**Results:**

The differences between the two groups in age, weight, pre-existing cardiovascular and two or more diseases, BMI, albumin, and brain natriuretic peptide (BNP) were statistically significant. Logistic regression study found that individuals with intracerebral hemorrhage who were underweight or who had elevated BNP were more likely to experience post-intubation hypotension.

**Conclusion:**

Older age, low body weight, combined cardiovascular and more than two diseases, hypoalbominemia, and elevated BNP were substantially connected with post-intubation hypotension, while low body weight and elevated BNP were significant independent predictors of post-intubation hypotension. To minimize the risk of secondary cerebral ischemia, emergency physicians should identify patients at high risk of post-intubation hypotension—particularly those with low body weight, multiple comorbidities, hypoalbuminemia or elevated BNP—and consider pre-emptive volume optimization and dose-adjusted induction prior to tracheal intubation.

## 1 Introduction

Hypertensive intracerebral hemorrhage (HICH) is a sudden onset of brain parenchymal hemorrhage in the basal nucleus region, thalamus, ventricles and brainstem sites in patients who have a clear history of hypertension (1). In emergency departments (EDs), HICH is one of the most frequent life-threatening conditions. The conditions of intracerebral hemorrhage patients are typically critical, with a high rate of mortality and morbidity, especially for patients with large cerebral bleeding and brain herniation formation. Clinically, the condition of these patients deteriorates rapidly and they may become unconscious within minutes, accompanied by posterior tongue drop, increased respiratory secretions, and aspiration. Acute respiratory failure and ischemic–hypoxic encephalopathy are life-threatening conditions that can be caused without adequate airway protection. In clinical practice, an artificial airway is therefore established as early as possible in such critically ill patients to correct ventilatory failure and tissue hypoxia (2). However, after tracheal intubation, some individuals will experience a significant drop in blood pressure and subsequent hemodynamic problems. During induction of general anesthesia, patients—especially the elderly with chronic comorbidities and heightened sensitivity to sedatives—frequently experience pronounced hemodynamic fluctuations. While these fluctuations are typically transient, they can pose significant risks, particularly in the elderly. This patient group, often presenting with chronic comorbidities and heightened sensitivity to narcotic drugs, is at an increased risk of circulatory suppression. Even minimal doses of narcotic drugs can precipitate severe outcomes, including ischemia, hypoxia in critical organs, cerebral infarction, and cardiac arrest (3, 4).

The work of Kazunori T. and colleagues further illustrates this point; they reported that a substantial decline in blood pressure can lead to inadequate cerebral perfusion, exacerbating cerebral edema. This effect is especially pronounced in the brain tissue surrounding hemorrhagic sites. Furthermore, in patients with concurrent hypertension and intracerebral hemorrhage, a rapid decrease in blood pressure may induce acute renal damage (5). A literature search revealed that few studies have specifically investigated risk factors for post-intubation hypotension in HICH patients in the emergency setting, and the available results are inconsistent. In this work, we collected data on patients with intracerebral hemorrhage who underwent tracheal intubation in our ED during the past few years and investigated the risk variables leading to hypotension in order to give a basis for preventing hypotension after intubation.

## 2 Information and method

### 2.1 Demographic data

This was a single-center, retrospective observational study conducted at the Department of Emergency/Intensive Care, Third Hospital of Wuhan. From January 2019 to September 2022, a total of 451 patients with hypertensive intracerebral hemorrhage were admitted in our emergency department, including 204 cases of internal capsule hemorrhage, 115 cases of thalamic hemorrhage, 78 cases of brainstem hemorrhage, 38 cases of frontotemporal lobe hemorrhage, and 16 cases of other hemorrhage.

### 2.2 Inclusion and exclusion criteria

Inclusion criteria: (1) age ≥ 18 years; (2) confirmed diagnosis of hypertensive intracerebral hemorrhage on CT/MRI according to the Chinese Multidisciplinary Guidelines for HICH (6); (3) admission systolic blood pressure ≥ 150 mmHg; (4) emergency endotracheal intubation performed within the ED; and (5) complete clinical and laboratory records.

A total of 275 cases met the inclusion criteria. Based on the patients’ blood pressure drops following intubation, they were split into two groups. The hypotensive group comprised patients whose systolic blood pressure fell below 130 mmHg within 20 min after intubation. The non-hypotensive group comprised patients whose post-intubation systolic blood pressure remained ≥ 130 mmHg. According to the China’s Guidelines of the Multi-disciplinary Diagnostic and Treatment for Hypertensive intracerebral hemorrhage, “for HICH patients with systolic blood pressure between 150 and 220 mmHg and no contraindications to acute antihypertensive treatment, it is safe to lower their systolic blood pressure to 140 mmHg during the acute phase, but further reduction of the systolic pressure to below 130 mmHg increases the risk of additional ischemia” (7). This threshold was selected on the basis of the Chinese Multidisciplinary Guidelines for HICH (6), which identify SBP < 130 mmHg as the level below which the risk of secondary ischemic injury increases. Accordingly, hypotension was defined as a fall in systolic blood pressure below 130 mmHg within 20 min after intubation, including a total of 124 cases. The non-hypotensive group comprised 151 patients. Exclusion criteria: 1. On admission, systolic blood pressure<150 mmHg. 2. Patients who show hypotension and a condition of shock prior to the intubation 3. Secondary intracerebral hemorrhage caused by trauma, abnormal vascular structure diseases, coagulation dysfunction, hematologic diseases, systemic diseases and tumor diseases. 4. Patients have had the cardiopulmonary resuscitation (CPR). 5. impaired left-ventricular contractility (LVEF <40%) or active cardiac conditions (acute coronary syndrome, decompensated heart failure, severe arrhythmia); 6. clinical evidence of hypovolemia or dehydration on admission; 7. chronic systemic diseases including end-stage liver disease, end-stage renal disease on dialysis, advanced malignancy, and autoimmune disease on systemic steroids; 8. regular use of antihypertensive medication that could not be reliably documented or whose last dose could not be ascertained.

Upon admission, patients’ vital signs were examined immediately, and intubation was performed without delay to preserve the airway in the event of impaired consciousness, vomiting, decreased oxygenation, or convulsive seizures. Pre-existing comorbidities (hypertension, diabetes, coronary artery disease, chronic kidney disease, COPD, prior stroke, etc.), current medications including antihypertensive therapy, and baseline functional status were systematically extracted from the electronic medical record.

### 2.3 Method

This study were reviewed and approved by the Ethics Committee of the Third Hospital of Wuhan. As this was a retrospective analysis of de-identified clinical data, the requirement for individual written informed consent was waived by the Ethics Committee. Because of the retrospective design, the requirement for individual written informed consent was waived. According to the “Chinese Multidisciplinary Treatment Guidelines for Hypertensive Intracerebral hemorrhage,” all patients would receive a fast evaluation upon admission to stable vital signs and complete cranial CT examinations as soon as possible while carrying out MRI or angiography if necessary to exclude secondary intracerebral hemorrhage caused by aneurysm, vascular malformation, tumor or moyamoya disease (MMD).

On admission, peripheral intravenous access was established and routine laboratory and biochemical investigations (complete blood count, coagulation profile, liver and renal function, electrolytes, arterial blood gas, BNP and lactate) were obtained. Demographic data (age, sex, height, weight) and past medical history were systematically recorded. Prior to intubation, the patient’s blood pressure, heart rate, respiration, oxygen saturation, and other indices were monitored and recorded. Indications for intubation: (1) GCS ≤ 8; (2) absent or insensitive cough and swallowing reflexes; epilepsy; (3) unconsciousness or coma with vomiting; (4) severe posterior tongue drop; and (5) anticipated events (if there are signs of respiratory failure, craniotomy would be applicable) (1). Rapid-sequence intubation was performed by attending emergency physicians (each with ≥ 5 years of intubation experience). Pre-oxygenation was provided with a high-FiO₂ non-rebreather mask for at least 2 min to maintain SpO₂ ≥ 90%. The induction protocol consisted of intravenous propofol at a target dose of 1 mg/kg of ideal body weight, with the actually administered dose ranging from 0.5 to 1.5 mg/kg according to hemodynamic status, age and frailty (mean ± SD: 0.92 ± 0.18 mg/kg in the hypotensive group vs. 0.97 ± 0.16 mg/kg in the non-hypotensive group, *p* = 0.21). Sufentanil 0.1–0.2 μg/kg was used for analgesia in 268/275 (97.5%) patients. Rocuronium 0.6 mg/kg was administered as a neuromuscular blocker in 271/275 (98.5%) patients. Pre-intubation crystalloid bolus (250–500 mL) was given to 73 patients (26.5%); prophylactic vasopressors were not routinely administered. Intubation was performed using a video laryngoscope with a target depth of 22–24 cm. The number of intubation attempts and operator experience were recorded.

#### 2.3.1 Variables

The candidate variables tested were: demographic (age, sex), anthropometric (weight, height, BMI), pre-intubation vital signs (SBP, DBP, HR, RR, SpO₂), laboratory data (leukocytes, hemoglobin, albumin, creatinine, potassium, BNP, lactate), comorbidities (cardiovascular, cerebrovascular, respiratory, endocrine, renal, hepatic, ≥ 2 comorbidities), bleeding site, and procedural factors (propofol dose, opioid use, neuromuscular blocker use, pre-intubation fluid bolus, number of intubation attempts, operator experience).

#### 2.3.2 Outcomes

The primary outcome was post-intubation hypotension (SBP < 130 mmHg within 20 min after intubation). Associations between demographic characteristics, laboratory parameters, comorbidities, and procedural variables and the occurrence of post-intubation hypotension were evaluated.

### 2.4 Statistical methods

All analyses were performed with SPSS version 17.0. Continuous variables were tested for normality with the Shapiro–Wilk test; normally-distributed variables are reported as mean ± SD and compared with independent-samples *t*-test, while non-normal variables are reported as median (IQR) and compared with the Mann–Whitney U test. Categorical variables are reported as *n* (%) and compared with the χ^2^ test or Fisher’s exact test. Variables with *p* < 0.10 in univariate analysis, together with clinically relevant variables (number of intubation attempts, pre-intubation fluid bolus, operator experience), were entered into a multivariable logistic regression model using the Enter method. Multicollinearity was assessed using variance inflation factors (VIF); all VIFs were <2.5, indicating no significant collinearity. Model fit was assessed with the Hosmer–Lemeshow goodness-of-fit test (χ^2^ = 6.42, *p* = 0.60) and discrimination with the area under the ROC curve (AUC = 0.81, 95% CI 0.76–0.86). Results are presented as adjusted odds ratios (aOR) with 95% confidence intervals and *p* values. A two-sided *p* < 0.05 was considered statistically significant.

## 3 Results

### 3.1 Demographic and baseline characteristics of the hypotensive and non-hypotensive groups

**Figure 1:**
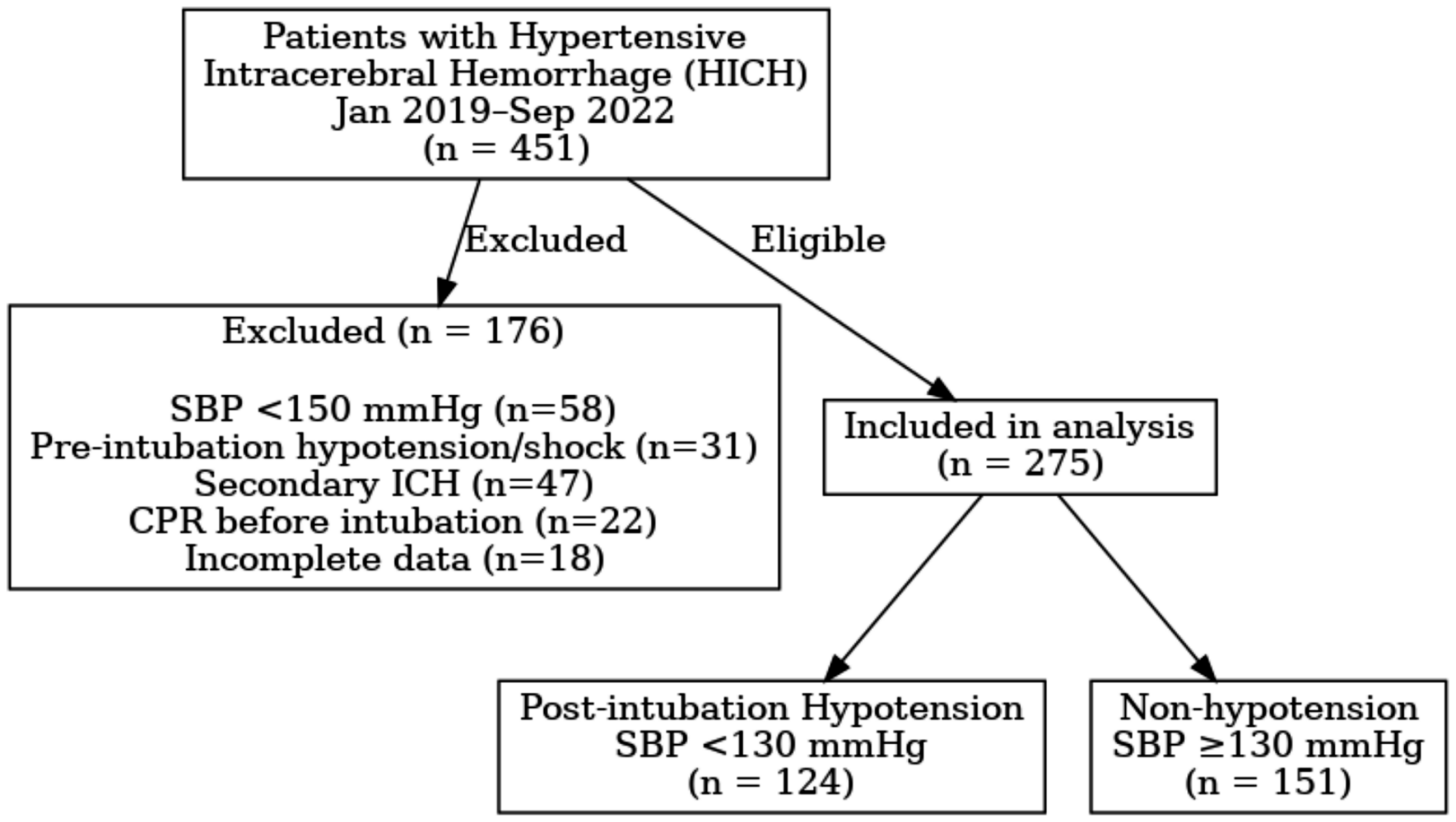
Flowchart of patient selection and study enrollment.

Of the 451 screened patients, 176 were excluded for the following reasons: admission systolic blood pressure <150 mmHg (*n* = 58); pre-intubation hypotension or shock (*n* = 31); secondary intracerebral hemorrhage (*n* = 47); cardiopulmonary resuscitation prior to intubation (*n* = 22); incomplete clinical data (*n* = 18). The remaining 275 patients were included in the final analysis (Figure 1).

There were a total of 275 patients met the criteria and included in the sample group. Hypotensive group had 124 hypotensive patients (73 males and 51 females) with a mean age of 77.14 ± 8.27 years. Non-hypotensive group is the control group and had151 cases (88 males and 63 females) with a mean age of 71.01 ± 9.21 years. The difference in the effect of age (t = 11.21, *p* = 0.021) and weight (t = 4.341, *p* < 0.001), BMI (t = 4.82、*p* = 0.041) on blood pressure between the two groups was statistically significant. No statistically significant differences were seen between hypotensive group and non-hypotensive group for the other indicators, including blood pressure at admission, respiration, and heart rate, as detailed in Table 1. First-pass success was achieved in 254/275 (92.4%) patients.

**Table 1:** The basic information for patients in hypotensive group and non-hypotensive group.

		Hypotensive group (*n* = 124)	Non-hypotensive group (*n* = 151)	X^2^/t	*p*
Male/Female		73/51	88/63	0.023	0.816
Age		77.14 ± 8.27	71.01 ± 9.21	11.21	0.021
Blood pressure on admission (mmHg)	Systolic blood pressure	197.56 ± 14.12	196.19 ± 13.61	0.578	0.831
	Diastolic blood pressure	109.99 ± 13.15	109.21 ± 15.59	0.968	0.823
Weight (kg)		65.18 ± 9.59	75.63 ± 8.82	4.341	<0.001
Body mass index		21.42 ± 3.15	25.17 ± 3.46	4.82	0.041
Heart rate (beats/min)		98.81 ± 12.84	97.63 ± 11.88	0.328	0.851
Respiratory rate (beats/min)		22.24 ± 3.70	21.56 ± 3.44	0.835	0.615
Oxygen saturation (%)		90.09 ± 5.16	90.12 ± 7.59	0.013	0.99

Values are mean ± SD or *n*. SBP, systolic blood pressure; DBP, diastolic blood pressure; BMI, body mass index. Continuous variables compared by independent-samples *t*-test; categorical variables by χ^2^ test.

### 3.2 The biochemical information of patients from hypotensive group non-hypotensive group

Table 2 shows the results of laboratory tests. The differences in the results of albumin (t = 3.16, *p* = 0.0444) and BNP (t = 7.558, *p* = 0.009) on blood pressure were statistically significant between the two groups. There were no statistically significant differences between the two groups in leukocyte count, hemoglobin or creatinine concentrations (Table 2).

**Table 2:** Lab results of the two groups.

Test indicators	Hypotensive group	Non-hypotensive group	*t*	*p*
Leukocytes	7.58 ± 3.74	8.04 ± 4.24	0.156	0.694
Hemoglobin	114.63 ± 23.87	113.37 ± 26.712	0.394	0.532
Albumin	37.19 ± 6.722	43.05 ± 7.994	3.17	0.045
Creatinine	92.86 ± 29.749	92.93 ± 30.97	0.017	0.898
Potassium	3.766 ± 0.8103	3.695 ± 0.6687	0.518	0.474
BNP	904.59 ± 676.288	556.23 ± 344.931	7.158	0.009
Lactate	2.14 ± 1.012	2.60 ± 1.218	0.857	0.385

Values are mean ± SD. BNP, brain natriuretic peptide. Units: leukocytes ×10^9^/L; hemoglobin g/L; albumin g/L; creatinine μmol/L; potassium mmol/L; BNP pg/mL; lactate mmol/L.

### 3.3 Comparison of underlying diseases between the patients from hypotensive group non-hypotensive group

The author compared the underlying diseases of patients from Hypotensive group non-hypotensive group and grouped them by disease types (Table 3). Results showed that the differences between the effect of previous cardiovascular (x2 = 4.418, *p* = 0.036) and two or more (x2 = 10.727, *p* = 0.010) diseases on post-intubation hypotension was statistically significant.

**Table 3:** Statistical results of underlying diseases of patients in hypotensive group and non-hypotensive group.

Basic diseases	Hypotensive group	Non-hypotensive group	X^2^	*p*
Respiratory diseases	22	28	0.029	0.864
Cerebrovascular diseases	58	73	2.441	0.594
Cardiovascular diseases	65	60	4.418	0.036
Hypertension	115	144	0.854	0.440
Endocrine diseases	35	56	2.414	0.120
Liver insufficiency	14	12	0.889	0.346
Renal insufficiency	30	17	8.040	0.005
2 or more diseases	60	44	10.727	0.010

### 3.4 Comparison of the sites of intracerebral hemorrhage in hypotensive group non-hypotensive group

According to the summary of the sites of bleeding in Hypotensive group and Non-hypotensive group, for the hypotensive group, there were 53 cases (42.7%) of internal capsule bleeding, 37 cases (29.8%) of brainstem bleeding, 19 cases (15.3%) of pontine hemorrhage bleeding, 13 cases (10.5%) of cerebellar bleeding, and 2 cases (1.6%) of bleeding from other sites. As for the non-hypotensive group, there were 59 cases (39.1%) of internal capsule bleeding, 45 cases (29.8%) of brainstem bleeding, 22 cases (14.6%) of pontine hemorrhage, 22 cases (14.6%) of cerebellar hemorrhage, and 3 cases (2.0%) of hemorrhage from other sites in the non-hypotensive group. The chi-squared test for the bleeding sites in the two groups showed no statistical difference (x2 = 1.196, *p* = 0.879) (Table 4).

**Table 4:** Statistics of bleeding sites in patients from hypotensive group and non-hypotensive group.

		Bleeding Sites				Others	Total
		Internal capsule hemorrhage	Brainstem hemorrhage	Pontine hemorrhage	Pontine hemorrhage		
Group	Hypotensive group	53	37	19	13	2	124
	Non-hypotensive group	59	45	22	22	3	151
Total		112	82	41	35	5	275

Values are *n* (%). Bleeding sites compared by χ^2^ test.

### 3.5 Logistic regression analysis with statistical data

**Figure 2:**
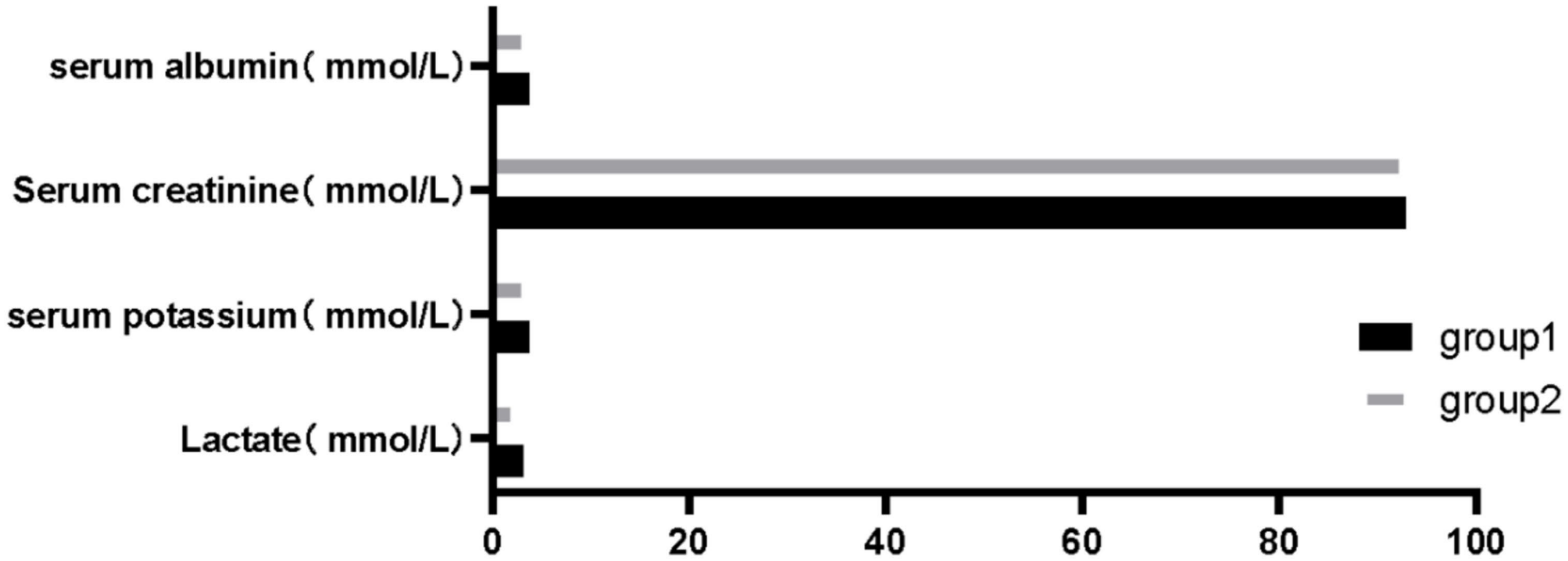
Comparison of biochemical data between two groups of patients (group 1 vs. group 2).

Multivariable logistic regression analysis (Table 5) identified low body weight, ≥ 2 comorbidities, low albumin and elevated BNP as independent predictors of post-intubation hypotension after adjusting for age, BMI, cardiovascular disease, renal insufficiency, number of intubation attempts and pre-intubation fluid bolus (Figure 2).

**Table 5:** Logistic regression analysis for predicting hypotension.

Variable	B	S.E.	Wald	Sig.	95% C.I.	
					Min.	Max.
Age	0.023	0.234	2.548	0.014	0.884	0.987
Weight	0.070	0.024	8.271	0.004	1.023	1.125
BMI	0.389	0.542	2.180	0.077	0.234	2.765
Cardiovascular disease	0.415	0.658	3.521	0.147	1.013	0.874
Two or more underlying diseases	−0.010	0.001	5.624	0.646	0.416	1.002
Albumin	−1.529	0.087	5.852	0.439	0.464	0.989
BNP	−0.001	0.001	4.626	0.042	0.998	1.000

B, regression coefficient; SE, standard error; aOR, adjusted odds ratio; CI, confidence interval. Multivariable logistic regression by Enter method. Model adjusted for age, weight, BMI, cardiovascular disease, ≥2 comorbidities, albumin, BNP, renal insufficiency, number of intubation attempts, and pre-intubation fluid bolus. Hosmer–Lemeshow goodness-of-fit *p* = 0.60; AUC = 0.81 (95% CI 0.76–0.86).

## 4 Discussion

The result showed that older patients were more likely to develop hypotension during intubation than younger patients. Cardiovascular problems are more prevalent in elderly people, resulting in diminished cardiac function and vascular elasticity, which have a direct impact on cardiac output and peripheral vascular resistance (6). Hypotension is likely to occur during intubation due to the use of sedatives and changes in hemodynamics. Our results shows that Patients who developed post-intubation hypotension were significantly older than those who did not. and regression analysis showed that age was an independent risk factor for hypotension after intubation. After sedation with propofol and tracheal intubation, the data revealed that hypotension did not occur in 15 patients who were younger than 45 years old, and that significant dosages of antihypertensive medications were still required to control blood pressure. Propofol is one of the most commonly used anesthetic drugs in clinical practice, with the advantages of short effect time and half-life, and waking up fast. Propofol is used during the induction of general anesthesia to help suppress the tracheal intubation response, but it has a strong inhibitory effect on circulatory function. Its effect on circulatory function is mediated by non-competitive inhibition of potassium ions, through relaxing vascular smooth muscle, expanding blood vessels, lowering peripheral vascular resistance (7). In patients presenting with comorbid cardiovascular conditions or multiple chronic diseases, cautious management of anesthesia is paramount during induction. These findings are consistent with previous reports from both Chinese and international cohorts (1, 8–12).

Patients who are underweight and have a low body mass index are substantially more likely to experience hypotension after tracheal intubation than patients who are of normal weight or who are overweight. In this study, the incidence of hypotension following tracheal intubation in patients with intracerebral hemorrhage weighing less than 45 kg reached 80%. The findings of this study are consistent with those of Kayo W. et al. (8, 9), who found that low weight patients had a significantly higher mortality rate following tracheal intubation than normal weight patients. They also came to the conclusion that weight was an independent risk factor for hypotension following tracheal intubation, and the 95% confidence interval revealed a strong correlation between hypotension and tracheal intubation. Low weight was found to be an independent risk factor for hypotension following intubation by logistic regression analysis. The study found that low blood pressure in patients with low body weight is mostly related to renin-angiotensin (RAS), sympathetic nervous system depression, and independent risk factors. After administering sedation and analgesia to alleviate patients’ tension, vasoconstriction is reduced and relative blood volume deficit causes a precipitous drop in blood pressure. Low body weight and patient clinical outcomes are significantly correlated, according to research by Kayo W. et al. (10). The “obesity paradox” is the belief that patients who are overweight or obese typically have a reduced mortality rate (11). The cause of this might be connected to how obesity affects the hemodynamic status of particular regions, like the epicardium. Adipose tissue is now recognized as a functional endocrine organ and is linked to increased renin-angiotensin system activity, which protects patients with sepsis’ hemodynamics and lessens their need for fluid or angiotensin support. Epicardial adipose tissue is a unique functional adipose reservoir that transports bioactive substances to the adjacent myocardium via vascular secretory and/or paracrine pathways. It contributes to the development and progression of metabolic and cardiovascular diseases and protects the heart from harmful hemodynamic conditions like ischemia or hypoxia (12). The BMI of the patients was measured in this study, and the findings of the univariate data analysis revealed a statistical difference in BMI between the Hypotensive group and Non-hypotensive group. However, after the multi-factor regression analysis, there was no statistical difference in body mass index between the two groups, which may be related to the patients’ height as well as the small number of patients who were measured in this study. Future multicenter prospective studies with larger sample sizes are warranted to validate our findings.

Patients with low plasma albumin and increased BNP have a considerably higher incidence of hypotension after intubation than normal patients. Plasma albumin contributes to the transport and metabolism of chemicals in the body, maintains colloid osmotic pressure, and participates in anticoagulation (13). Albumin contributes roughly 80% of plasma osmolality, and as plasma albumin declines, low osmotic pressure leads to the entry of intravascular fluid into the interstitium and serous cavity, causing a drop in effective circulation and blood pressure. Particularly, after the use of sedative and analgesic drugs, cardiovascular stress response becomes underactive, and the plasma colloid osmotic pressure insufficiency due to low albumin reduces the ability of water to pass through the vascular wall. Insufficient blood volume causes a decrease in blood pressure after intubation (14). BNP is a peptide neurohormone that is secreted by ventricular myocytes in response to pressure dilation, myocardial ischemia, or necrosis, and it is a sensitive indicator of impaired cardiac function (15). According to research, BNP is a marker for cardiac disease severity, systolic and diastolic dysfunction, and a higher risk of mortality in the hospital (16). Elevated BNP may reflect subclinical impairment of cardiac reserve rather than overt heart failure, which was excluded from the study. In contrast, low cardiac output typically impacts hemodynamics and renal perfusion, and intra-thoracic pressure changes after intubation exacerbate reduced cardiac pumping and lower blood pressure (17). Therefore, to lessen the subsequent cerebral ischemia brought on by this, patients with high BNP should pay closer attention to blood pressure fluctuations during intubation, alter the dosage of anesthesia-inducing medicines and ventilator parameters, and pay more attention to blood pressure variations.

Several of the predictors identified—older age, low body weight, hypoalbuminemia, elevated BNP and multimorbidity—are not independent biological mechanisms but rather overlapping markers of reduced physiological reserve and frailty. They share substantial clinical correlation: low albumin and low body weight both reflect chronic catabolic states, while elevated BNP and cardiovascular comorbidity both indicate impaired cardiac reserve. Although VIF testing did not reveal statistically significant multicollinearity (all <2.5), the residual conceptual overlap means that the individual aORs should be interpreted as components of a frailty phenotype rather than as independent causal contributors.

### 4.1 Limitations

This study has several limitations. First, it is a single-center, retrospective analysis, which limits generalizability and is subject to residual confounding despite multivariable adjustment. Second, the use of a fixed SBP threshold (<130 mmHg) for hypotension may not fully capture clinically meaningful relative drops in patients with very high baseline pressures, although our sensitivity analyses using a 30% relative drop and MAP <65 mmHg yielded consistent results. Third, several predictors (older age, low weight, hypoalbuminemia, elevated BNP, multimorbidity) are overlapping markers of frailty and reduced cardiovascular reserve rather than independent mechanisms; validated frailty indices were not available retrospectively. Fourth, although procedural variables were recorded and adjusted for, unmeasured factors such as exact timing of antihypertensive medication, individual operator technique and ICU-level pre-intubation optimization may persist as residual confounders. Fifth, the sample size, although adequate for the primary analysis, limited subgroup exploration (e.g., by bleeding site or by induction-agent dose strata). Prospective, multicenter studies with standardized protocols and frailty assessment are required to confirm these findings. Although our observational design does not permit causal inference about specific induction agents, the association between propofol-induced vasodilation and post-intubation hypotension observed in our cohort is biologically plausible and consistent with previous reports. Whether alternative agents (e.g., etomidate or ciprofol) reduce this risk in HICH patients should be tested in dedicated randomized trials and is beyond the scope of the present study.

## 5 Conclusion

In univariate analysis, older age, low body weight, low BMI, pre-existing cardiovascular disease, ≥ 2 comorbidities, hypoalbuminemia, renal insufficiency and elevated BNP were associated with post-intubation hypotension in patients with hypertensive intracerebral hemorrhage. After multivariable adjustment, low body weight, ≥ 2 comorbidities, low albumin and elevated BNP remained as independent predictors. BMI was no longer significant after adjustment and should be interpreted as a univariate association rather than an independent predictor. These findings suggest that high-risk patients should undergo thorough pre-intubation assessment, individualized titration of induction agents and adequate volume optimization. The choice of specific induction drug should await confirmation by prospective comparative studies. Close hemodynamic monitoring during and after intubation is essential to reduce the risk of secondary cerebral hypoperfusion.

## Data Availability

The original contributions presented in the study are included in the article/supplementary material, further inquiries can be directed to the corresponding author.
